# Stereotactic body radiation therapy is beneficial for a subgroup of patients with urothelial cancer and solitary metastatic disease: a single institution real-world experience

**DOI:** 10.1186/s13014-024-02465-y

**Published:** 2024-06-16

**Authors:** Fernanda Costa Svedman, Karin Holmsten, Faith Jawdat, Wehazit Hailom, Daniel Alm, Vitali Grozman, Anders Ullén

**Affiliations:** 1 Department of Oncology-Pathology, Karolinska Institute, Stockholm, Sweden; 2 Department of Pelvic Cancer, Genitourinary Oncology and Urology Unit, Theme Cancer, Karolinska University Hospital, Stockholm, 171 76 Sweden; 3 Department of Oncology, Capio S:t Göran Hospital, Stockholm, Sweden; 4 Department of Radiation Oncology, Karolinska University Hospital, Stockholm, Sweden; 5 Department of Radiology, Karolinska University Hospital, Stockholm, Sweden; 6 Department of Molecular Medicine and Surgery, Karolinska Institute, Stockholm, Sweden

**Keywords:** Stereotactic body radiation therapy, SBRT, Oligometastatic, Oligoprogressive, Urothelial cancer

## Abstract

**Background:**

Standard treatment options for patients with metastatic urothelial cancer (mUC) include systemic platinum-based chemotherapy, immunotherapy, antibody-drug-conjugates, and targeted therapy. Oligometastatic disease (OMD) may be an intermediate state between localized and generalized cancer. The best treatment strategy for OMD and oligoprogressive (OPD) disease is poorly studied in mUC but local stereotactic body radiation therapy (SBRT) could be an option to avoid or delay systemic treatment. The aim of this study was to assess the efficacy and feasibility of SBRT given in a real-world patient population.

**Methods:**

All patients with mUC treated with SBRT at Karolinska University Hospital, Stockholm, Sweden between 2009 and 2022 were included in this study. Baseline clinical characteristics, treatment data, SBRT dosimetry data and treatment outcome were collected retrospectively. The study endpoints were local control rate (LCR), progression-free-survival (PFS), overall survival (OS) and feasibility of SBRT.

**Results:**

In total 39 patients were treated with SBRT. The median follow-up was 25.6 months. The LCR was 82%. PFS and OS were 4.1 and 26.2 months, respectively. Treatment was well tolerated; all patients but one (treatment related pain) completed the planned SBRT. Number of metastases irradiated with SBRT was significantly associated with outcome; patients with only one irradiated lesion had more favourable PFS compared to individuals with 2 or more metastases (HR 4.12, 95% CI: 1.81–9.38, *p* = 0.001). A subgroup of patients (15%) achieved a sustained long-term survival benefit and never required systemic treatments after SBRT.

**Conclusions:**

SBRT was well tolerated and associated with high LCR. A subpopulation of patients with single metastatic lesion achieved long-term OS and never required subsequent systemic treatment after SBRT. Prospective randomized studies are warranted to discover treatment predictive biomarkers and to investigate the role of SBRT in oligometastatic UC.

**Supplementary Information:**

The online version contains supplementary material available at 10.1186/s13014-024-02465-y.

## Background

Bladder cancer is among the 10 most common cancers worldwide causing more than 210 000 deaths globally in 2020 [1]. Affected patients are predominantly male between 65 and 70 years old, seeking health care due to haematuria [2].

The recommended first line therapy for metastatic urothelial cancer (mUC) is 4–6 cycles of platinum-based chemotherapy followed by maintenance immunotherapy for those patients achieving disease control with chemotherapy. The prognosis is however poor with a median progression-free survival (PFS) of only 4 months and a median overall survival (OS) of 24 months. Moreover, the incidence of grade ≥ 3 adverse events is almost 50% [3, 4]. Although the treatment scenario for patients with metastatic urothelial cancer is developing quickly with new drugs, such as antibody drug conjugates and targeted therapy, the prognosis remains dismal and novel treatment strategies an unmet medical need [5–7].

In 1995 Hellman and Weichselbaum suggested an intermediate state between localized and metastatic disease, namely oligometastatic disease (OMD) [8]. The practical significance is that patients with few metastases can receive potentially curative local treatment approaches instead of palliative systemic treatment [8]. Radiation therapy is an attractive approach for local treatment given it is non-invasive compared to surgery. Defining OMD is complex, but the European Society for Radiation Oncology (ESTRO) and the American Society of Radiation Oncology (ASTRO) consensus suggests that a cancer patient with up to 5 metastases has OMD if all the metastatic sites can be safely treatable [9].

To the best of our knowledge, there is no data from prospective randomized phase 3 studies exploring the best treatment option for patients with urothelial cancers and OMD/oligoprogressive disease (OPD). A meta-analysis including 17 studies and 412 patients investigated the role of metastasectomy in mUC. The study showed a significantly more favourable OS for individuals treated with metastasectomy compared with non-operated patients. However, the studies included in this meta-analysis were mainly non-interventional and retrospective [10].

The role of stereotactic body radiation therapy (SBRT) in patients with mUC with OMD/ OPD is poorly investigated. Therefore, we performed a retrospective study in a single centre real-world population to evaluate local control rate (LCR), PFS, OS and feasibility of SBRT.

## Methods

Medical records from patients with mUC treated with SBRT at Karolinska University Hospital between 2009 and 2022 were retrospectively reviewed. Baseline clinical characteristics, treatments patterns before and after SBRT and clinic outcome after SBRT were collected. Further, information about the indication of SBRT was collected, i.e., primary OMD or OPD. OMD was defined according to above mentioned ESTRO/ASCO consensus criteria whereas the term OPD was used when most metastasis had shown good response to systemic treatment, but with few lesions (≤ 5 metastasis) progressing.

Patients receiving SBRT to brain metastases were excluded from the study due to poor life expectancy in patients with central nervous system involvement. If patients received sequential SBRT to treat new metastases, only the first SBRT therapy was considered in this analysis.

A radiation therapist reviewed dosimetry data of the given SBRT, e.g., target definition, treatment dates, prescribed total dose in Gray (Gy), number of fractions, clinical tumour volume (CTV) planning target volume (PTV) and the biologically effective dose (BED 10) for CTV and PTV.

Response evaluation was based on computerized tomography. A radiologist specialized on cancer imaging and post-radiotherapy evaluation retrospectively analysed all radiological exams from baseline until progressive disease and assessed local control rate, and local and systemic PFS.

The study was carried out following Good Clinical Practice, the Declaration of Helsinki and ethical approval was obtained from Stockholm Regional Ethics Committee, Sweden.

### Endpoints

Local control rate (LCR) was defined whether the patients had local progression within the SBRT-irradiated field or not. Local progression-free survival (local PFS) was defined as time from start of first SBRT to local progression or death, whichever came first. Data concerning LCR was calculated both per patient (*n* = 39) and per SBRT-irradiated lesion (*n* = 51). Systemic progression-free survival (PFS) was defined as time from of first SBRT to progress outside the SBRT-irradiated region or death, whichever came first. OS was defined as time from start of SBRT to death from any cause.

### Statistical analysis

To analyse nominal data Pearson χ2 test was used with a significance level of *p* < 0.05. Local PFS, PFS and OS were calculated using the log-rank (Mantel-Cox) method and visualized by using Kaplan-Meier survival curves. For survival analyses, adjustment for baseline parameters were done using univariable Cox-proportional hazards (CoxPH) regression. Hazard ratios (HR) were estimated with 95% CIs. Data were analysed using SPSS statistics software for Windows (version 26; IBM SPSS, Armonk, NY, USA).

## Results

### Baseline patient’s characteristics

In our cohort (*n* = 39) we identified 31 males (79%) and eight females (21%) (Table 1). The median age was 72 years-old (range 54–88 years old) and most subjects (95%) had Eastern Cooperative Oncology Group (ECOG) performance status (PS) 0–1. The primary tumour location was in the urinary bladder in 25 patients (64%) whereas in 14 subjects (36%) the primary tumour was in the upper urinary tract (UTUC). Thirty-four patients (87%) had been initially treated with curative intention prior the SBRT whereas five were diagnosed with de novo non-operable locally advanced or metastatic disease. Only 36% had received palliative systemic treatment before SBRT.

**Table 1:** Baseline patient characteristics

Patient variable	*n* = 39
Sex, *n* (%)	
Male Female	31 (79) 8 (21)
Age, yr median (range)	72 (54–88)
Age interval, years (%)	
54–67 68–75 76–88	12 (31) 13 (33) 14 (36)
ECOG performance status, *n* (%)	
0 1 2	21 (54) 16 (41) 2 (5)
Primary tumour location, *n* (%)	
Upper tract tumour Bladder	14 (36) 25 (64)
Primary curative treatments, *n* (%) Curative cystectomy or nephrectomy^a^ Radiotherapy Peri-operative chemotherapy^b^	34 (87) 30 (77) 4 (10) 10 (26)
Primary metastatic disease, *n* (%)	5 (13)
Palliative systemic treatment pre-SBRT, *n* (%)	14 (36)

^a^Additional three patients underwent palliative cystectomy or nephrectomy.

^b^Neo-adjuvant chemotherapy, *n* = 9, adjuvant chemotherapy, *n* = 1.

*ECOG* Eastern Cooperative Oncology Group.

### Treatment characteristics regarding SBRT

In total 51 metastatic lesions were treated with SBRT in 39 patients (Table 2). The most common metastatic sites of the first SBRT were lungs (45%) and lymph nodes (22%). Most patients had only one radiological metastasis at first SBRT (74%) but 26% of cases received SBRT against more than one lesion simultaneously. The indication of SBRT was primarily OMD in most individuals (69%). The median time between the diagnosis of muscle invasive urothelial cancer and the first SBRT was 23 months (range between 5 and 70 months). Summarized information about SBRT dosimetry including number of fractions, mean doses, and BED 10 against CTV and PTV is described in Table 2 with extended dosimetry data available in Supplementary Table 1. 63% of patients received palliative systemic treatment due to disease progression after SBRT and 32% were treated with further radiation, including three patients receiving sequential SBRT (Table 2).

**Table 2:** Treatment characteristics regarding SBRT

Variable	*n* = 39
Intention of first SBRT^a^, *n* (%)	
Oligometastatic disease (OMD) Oligoprogressive disease (OPD)	27 (69) 12 (31)
Metastatic site of first SBRT^a, b^, *n* (%)	
Local recurrence Lgll Bone Liver Lung Other^c^	2 (4) 11 (22) 6 (12) 4 (8) 23 (45) 5 (10)
Nr of metastases treated at first SBRT^a, b^, *n* (%)	
1 2 3 4	29 (74) 9 (23) 0 1 (3)
SBRT dosimetry data	
Fractions, nr median (range) Dose CTV mean, Gy median (range) Dose PTV mean, Gy median (range) Dose CTV mean BED10, Gy median (range) Dose PTV mean BED10, Gy median (range)	5 (2–10) 64 (35–75) 57 (31–68) 168 (65–210) 146 (55–183)
Palliative systemic treatment post-SBRT, *n* (%)	24 (62)
Palliative radiotherapy post-SBRT^d^, *n* (%)	12 (31)
No systemic palliative treatment post-SBRT and OS > 36 months^e^, *n* (%)	6 (15)

^a^First SRT treatment, three patients received consecutive SRT treatment

^b^Total nr of treated lesions was 51, in 39 patients

^c^Adrenal gland, *n* = 3, subcutaneous, *n* = 2

^d^Conventional radiotherapy or consecutive SRT

^e^One patient with consecutive SBRT

*SBRT* stereotactic body radiation, *CTV* clinical planning target volume, *Gy* Grey, *PTV* planning target volume, *BED* biological equivalent dose.

### Local control rate, survival, and tolerability

**Fig. 1:**
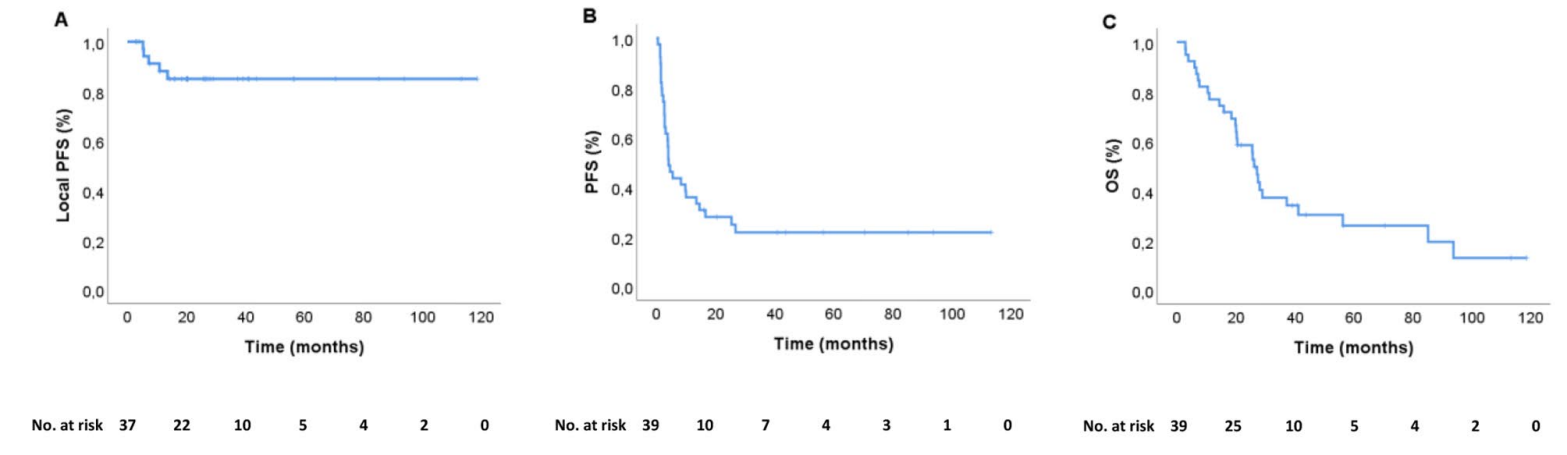
Kaplan Meier plots of **A**) local progression-free survival (local PFS) **B**) systemic progression-free (PFS) survival and C) overall survival (OS)

The median follow-up was 25.6 months (range 2.9–118.6). The LCR at 2 years was 85% (Fig. 1A) and only 6 of the SBRT irradiated lesions progressed locally. The pre-planned SBRT fractions were given to all patients except for one case where the SBRT was interrupted prematurely due to treatment related pain in the chest wall. There were no significant differences in LCR or local PFS regarding CTV and/or PTV-doses (Supplementary Table 2).

**Fig. 2:**
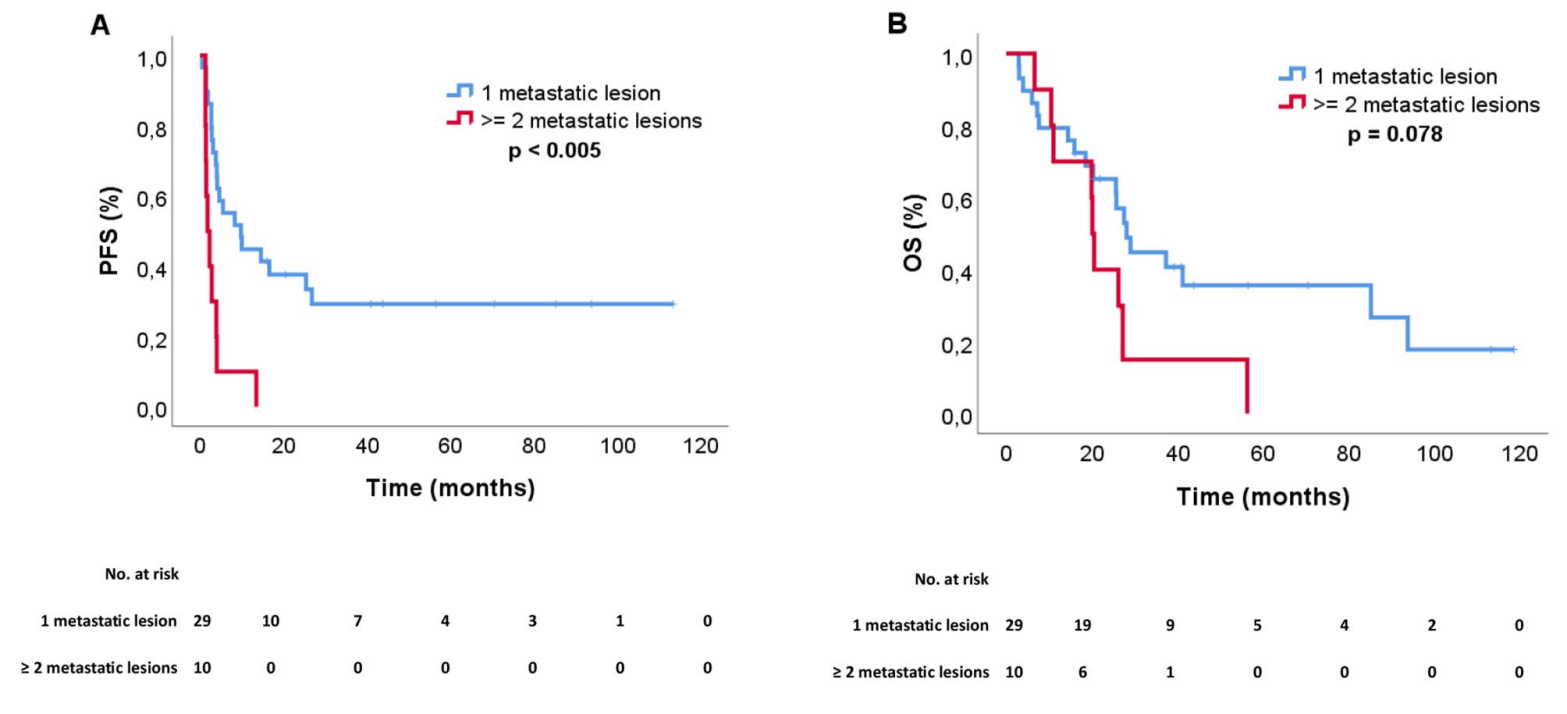
(**A**) Systemic progression-free survival (PFS) and (**B**) overall survival (OS) according to the number of metastases per patient treated with SBRT (1 vs 2 or more)

The median systemic PFS and OS were 4.1 and 26.2 months, respectively (Fig. 1B C). Patients with two or more metastatic lesions treated with SBRT simultaneously had a significantly shorter median systemic PFS of 1.8 month compared to patients with only one lesion treated, 9.8 months (HR 4.12, 95% CI: 1.81–9.38, *p* = 0.001) (Fig. 2A). There was a numerical difference in OS between these groups, with a median OS of 20.1 months versus 28.1, respectively however not statistically significant (HR 2.08, 95% CI: 0.91–4.76, *p* = 0.084) (Fig. 2B).

Univariate analyses were performed to investigate whether sex, age, ECOG PS, primary tumour location, primary curative treatment vs. primary metastatic disease, palliative systemic treatment pre-SBRT, location of SBRT-irradiated metastases, or number of metastases receiving SBRT were associated with PFS and OS. Palliative systemic treatment given pre-SBRT, and the number of metastases treated with SBRT were associated with survival outcome (Supplementary Table 3).

### Subgroups of patients with benefit of SBRT

**Fig. 3:**
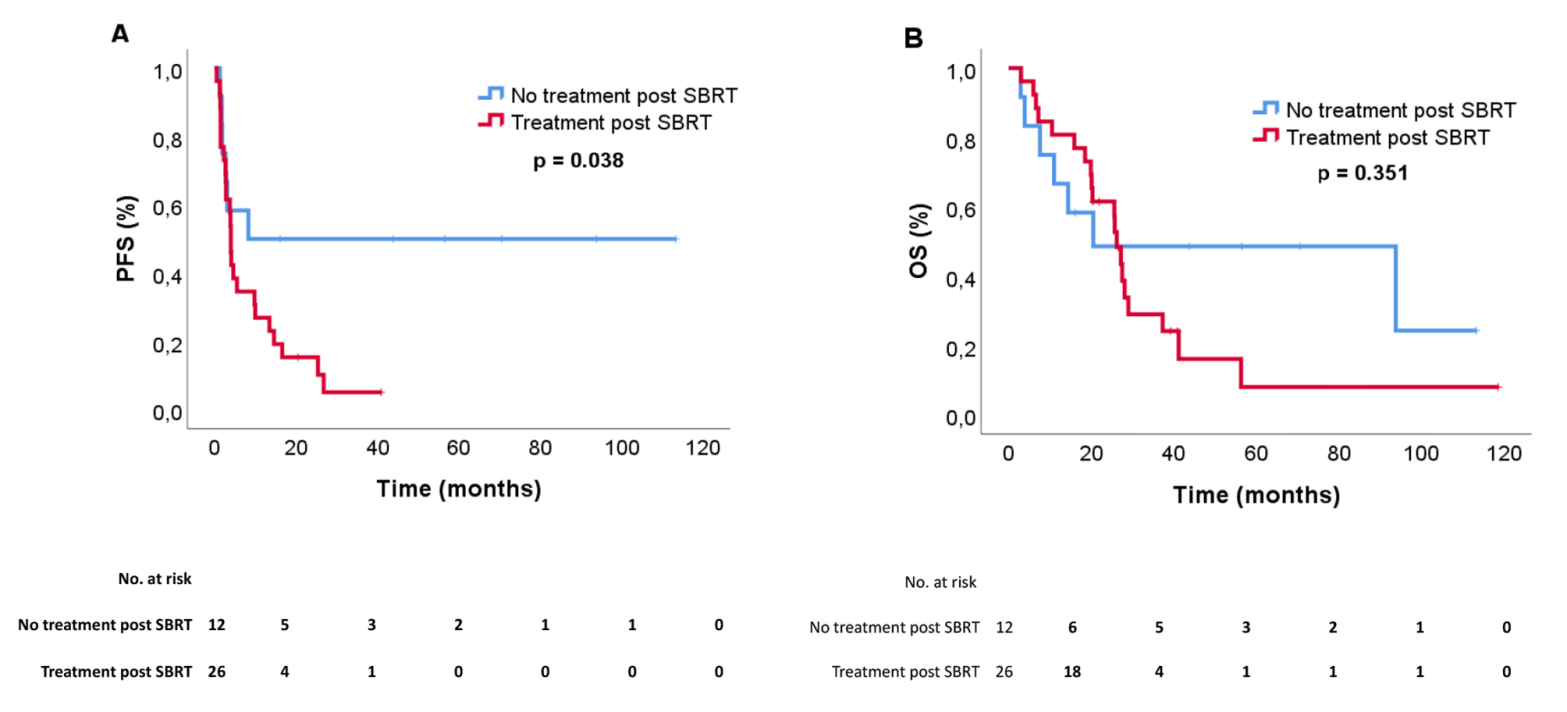
(**A**) Systemic progression-free survival (PFS) and (**B**) overall survival (OS) according to subsequent treatment (systemic and/or radiation treatment) post SBRT-treatment

Patients who did not receive any subsequent treatment after SBRT, neither systemic nor radiation treatment, had a significantly longer PFS of 8.3 months versus 4.0 months for patients who received treatment post-SBRT (HR 0.40, 95% CI: 0.16–0.98, *p* = 0.046) (Fig. 3A). For OS the KM-curves separate but were not statistically significant performing survival analyses (HR 0.66, 95% CI: 0.27–1.60, *p* = 0.353) (Fig. 3B).

An interesting subgroup of patients (*n* = 6, 15%) achieved long OS and never required systemic therapy after SBRT, whereof all of them had only one metastatic lesion. The OS for these individuals ranged between 43.8 and 113.3 months at study cut-off, and the only death in this sub-group was not cancer related (OS 93.8 months). Noteworthy, one of these patients received sequential SBRT due to disease progression to in total four different targets, inclusive in the brain, at different time points. This patient had a short systemic PFS of 2,7 months but was still alive at study cut-off with an OS of 118.6 months. All other patients never presented with recurrent disease.

## Discussion

This retrospective study was performed in a real-world cohort of patients with mUC that had received SBRT to treat either OMD or OPD. Our aim was to evaluate LCR, PFS, OS and feasibility of SBRT. A high LCR was achieved and the planned SBRT could be completed in all patients, except for one case with radiation related pain in the chest wall. The median PFS and OS were around 4 and 26 months, respectively. Notably, we found a subgroup of patients (15%) achieving durable survival of at least 42 months and never requiring systemic treatment after SBRT, even though one of these subjects received subsequential SBRT at different time points due to disease progression (including against brain metastases). However, most of the patients were subsequentially treated with systemic therapy due to further disease progression after SBRT.

In the present cohort most patients were older male with good ECOG PS and with the primary tumour originating from the urinary bladder. In most cases the treatment intention was to eradicate OMD in the lungs and lymph nodes. Only a minority of patients had primarily metastatic disease at diagnosis and the median time between the diagnosis of muscle invasive urothelial cancer and SBRT was 23 months. Patients with only one metastasis had a statistically significant better prognosis than those with two or more metastasis.

SBRT in patients with oligometastatic mUC is poorly studied and to the best of our knowledge there is no prospective randomized study evaluating this therapeutic option. A systematic review and meta-analysis including available reports addressing SBRT in patients with oligometastatic and oligoprogressive urothelial cancer identified 6 studies with 158 patients included in total [11]. The publications included in the meta-analysis come from small and retrospective studies with significant variation regarding inclusion criteria, assessment of outcomes, etc [12–17]. , making a comparison between these studies challenging. This meta-analysis showed that most patients were older males with primary tumours in the urinary bladder, in agreement with our data, and as expected for urothelial cancer [11]. The most common site of SBRT treated metastases were in the lymph nodes (52%), whereas in our cohort metastases were located mainly in the lungs (44%). Our outcomes of interest are within what has been reported in the meta-analysis. The LCR variated between 57% and 90%, median PFS between 3 and 10 months and median OS between 15 and 51 months [11].

A Canadian prospective, non-randomised study included 137 patients with different solid tumours (including genito-urinary) and extracranial OMD suitable for local approach, and with at least one lesion able to be treated with SBRT. 78% of the patients had only one metastatic lesion and the median follow-up was 36 months. The authors observed a plateau in the PFS curve at 36 months of around 18% similarly to our findings in the present study, indicating that there is a subpopulation of patients which achieves good clinical benefit of the given SBRT. The cumulative local progression rate at 36 months was 38% and PFS was accompanied by preservation of quality of life, good symptom control and less need of systemic therapy [18].

SBRT has been shown to be a beneficial therapeutic option in patients with other solid tumours as well, including renal cell cancer and lung cancer. The local tumour control rate has been reported high, SBRT was well tolerated, and some patients achieved a long-term clinical benefit [19, 20].

The main limitation of our study is the retrospective nature of the data without a control arm. Furthermore, the cohort is small and heterogenous regarding primary tumour location, distribution pattern of metastases, and previous treatments. However, the data is generated from real-world patients with a long follow-up and indicates that selected patients with solitary metastases may achieve long term OS without the need of subsequent systemic therapy.

Our cohort includes patients treated between 2009 and 2022 and many drugs, including immunotherapy, have been approved in this field since then and there is more to come [3, 5–7, 21, 22]. Toxicities and costs of these new regimens are nonetheless significant and delaying or avoiding systemic therapy may be beneficial and cost saving. In addition, OPD during immunotherapy is a well-known phenomenon and there is evidence that local therapy against OPD may result in durable disease control [23]. Besides, Daro-Faye et al. have demonstrated that radiation and immunotherapy may have synergistic anti-tumoral response in urothelial cancer [24] and emphasize the great need of more contemporary research in this field.

## Conclusion

SBRT can be an effective and feasible therapeutic option to achieve local tumour control in oligometastatic/oligoprogressive UC. Systemic cancer treatment may be postponed and sometimes completely avoided by use of SBRT in a subgroup of patients with single metastatic UC. On the contrary, OMD with 2 to 5 metastases may rather represent a disseminated phenotype for which systemic treatment should be considered upfront. However, prospective randomized trials using novel imaging- and biomarker approaches to improve patient selection to SBRT should be a priority for future studies.

### Electronic supplementary material

Below is the link to the electronic supplementary material.

Supplementary Material 1

## Data Availability

No datasets were generated or analysed during the current study.

## Abbreviations

ASTRO: American Society of Radiation Oncology
BED: biologically effective dose
CoxPH: Cox-proportional hazard
CTV: clinical tumour volume
ECOG: Eastern Cooperative Oncology Group
ESTRO: European Society for Radiation Oncology
Gy: Gray
HR: Hazard ratio
LCR: local control rate
mUC: metastatic urothelial cancer
OMD: oligo metastatic disease
OPD: oligoprogressive disease
OS: overall survival
PFS: progression-free survival
PS: performance status
PTV: planning target volume
SBRT: stereotactic body radiation therapy
UTUC: upper tract urothelial cancer
