# Supplementary material for: Stereotactic body radiation therapy is beneficial for a subgroup of patients with urothelial cancer and solitary metastatic disease: a single institution real-world experience
Source: Radiat Oncol. 2024 Jun 16;19:74. doi: 10.1186/s13014-024-02465-y (PMC11181669; doi:10.1186/s13014-024-02465-y)
Supplement: Supplementary file 1 — Supplementary Material 1 [file 13014_2024_2465_MOESM1_ESM.docx]

| **Supplementary Table 1** Extended dosimetric data | | | | | | | | | | | | | | |  |
| --- | --- | --- | --- | --- | --- | --- | --- | --- | --- | --- | --- | --- | --- | --- | --- |
| **Pat nr** | **Target** | **Start date SBRT**  **(Year-Month-Day)** | **Prescribed total dos (Gy)** | **Number of fractions** | **CTV**  **min (Gy)** | **CTV max (Gy)** | **CTV mean (Gy)** | **PTV**  **min (Gy)** | **PTV mean (Gy)** | **CTV min BED (Gy)** | **CTV max BED10 (Gy)** | **CTV mean BED10 (Gy)** | **PTV min BED10 (Gy)** | **PTV mean BED10 (Gy)** | |
| 2 | Lymph node para-aortic left | 2013-07-25 | 56 | 8 | 40,2 | 84,1 | 74,5 | 25,3 | 66,8 | 60,3 | 172,5 | 143,9 | 33,3 | 122,6 | |
| 3 | Lymph nodes para-aortic left + right | 2013-04-25 | 50 | 5 | 52,8 | 73,9 | 66,9 | 24,8 | 61,5 | 108,6 | 183,1 | 156,3 | 37,1 | 137,2 | |
| 4 | Lung right | 2016-12-14 | 45 | 3 | 55,7 | 67,5 | 63,6 | 37,2 | 56,3 | 159,3 | 219,3 | 198,6 | 83,5 | 162,2 | |
| 5 | Lung right | 2013-08-13 | 45 | 3 | 58,4 | 67,8 | 65,1 | 39,9 | 59,6 | 172,2 | 221,1 | 206,6 | 93,1 | 178,2 | |
| 5 | Liver | 2013-08-13 | 45 | 3 | 58,9 | 64,7 | 63,5 | 34,2 | 58,9 | 174,8 | 204,2 | 197,8 | 73,1 | 174,5 | |
| 6 | Lung right nr 1 | 2013-03-06 | 45 | 3 | 53,4 | 68,5 | 64,0 | 36,6 | 56,6 | 148,7 | 225,1 | 200,5 | 81,4 | 163,5 | |
| 6 | Lung right nr 2 | 2013-03-06 | 45 | 3 | 50,6 | 68,2 | 63,5 | 31,5 | 56,3 | 135,8 | 223,2 | 197,7 | 64,5 | 161,9 | |
| 6 | Lung left nr 1 | 2013-03-06 | 45 | 3 | 59,2 | 67,8 | 65,2 | 33,0 | 58,5 | 176,0 | 220,9 | 207,1 | 69,3 | 172,4 | |
| 6 | Lung left nr 2 | 2013-03-06 | 45 | 3 | 59,6 | 66,1 | 64,4 | 40,0 | 58,9 | 177,9 | 211,7 | 202,5 | 93,3 | 174,5 | |
| 7 | Local recurrence retroperitoneally | 2015-02-27 | 24 | 4 | 26,0 | 35,8 | 34,6 | 16,2 | 31,2 | 42,8 | 67,8 | 64,5 | 22,8 | 55,4 | |
| 8 | Adrenal gland right | 2012-11-21 | 45 | 3 | 57,2 | 67,9 | 64,2 | 37,6 | 60,5 | 166,3 | 221,5 | 201,3 | 84,7 | 182,6 | |
| 8 | Lymph node para-aortic left | 2012-11-21 | 45 | 3 | 60,4 | 67,1 | 65,2 | 39,7 | 59,5 | 182,0 | 217,4 | 206,9 | 92,4 | 177,4 | |
| 9 | Lung left | 2013-03-04 | 45 | 3 | 53,4 | 65,8 | 62,4 | 38,0 | 57,1 | 148,5 | 210,1 | 192,0 | 86,2 | 165,5 | |
| 10 | Liver | 2017-11-06 | 40 | 5 | 46,3 | 60,0 | 58,3 | 27,5 | 54,0 | 89,1 | 132,0 | 126,3 | 42,7 | 112,5 | |
| 11 | Lung left | 2013-03-07 | 45 | 3 | 58,2 | 68,7 | 64,3 | 20,7 | 56,5 | 170,9 | 226,0 | 202,3 | 34,9 | 162,8 | |
| 12 | Lung right | 2010-08-13 | 30 | 2 | 36,7 | 45,1 | 43,4 | 22,2 | 38,8 | 103,9 | 147,0 | 137,5 | 46,9 | 114,1 | |
| 13 | Adrenal gland right | 2014-07-17 | 56 | 8 | 35,6 | 82,8 | 72,5 | 21,5 | 65,4 | 51,4 | 168,6 | 138,3 | 27,2 | 118,8 | |
| 14 | Lung right | 2017-06-13 | 45 | 3 | 60,2 | 66,9 | 64,8 | 36,9 | 59,1 | 181,1 | 216,3 | 204,9 | 82,1 | 175,4 | |
| 15 | Local recurrence left kidney bed | 2018-05-03 | 50 | 5 | 34,1 | 66,9 | 62,5 | 25,1 | 55,2 | 57,4 | 156,6 | 140,6 | 37,7 | 116,2 | |
| 16 | Thoracic vertebra nr nine + soft tissue | 2018-04-04 | 50 | 5 | 20,8 | 57,2 | 42,3 | - | - | 29,4 | 122,7 | 78,0 | - | - | |
| 16 | Ramus inferior os pubis right | 2018-04-04 | 40 | 5 | 47,9 | 50,8 | 49,8 | 39,4 | 47,9 | 93,8 | 102,3 | 99,4 | 70,4 | 93,7 | |
| 17 | Lung left | 2018-05-14 | 45 | 3 | 60,1 | 67,0 | 64,7 | 40,3 | 59,9 | 180,5 | 216,4 | 204,4 | 94,6 | 179,5 | |
| 18 | Lung right | 2015-09-21 | 45 | 3 | 56,3 | 67,6 | 63,3 | 41,3 | 57,2 | 162,0 | 219,8 | 196,6 | 98,2 | 166,4 | |
| 18 | Lung left | 2015-09-21 | 45 | 3 | 55,2 | 66,6 | 60,2 | 41,7 | 55,0 | 156,9 | 214,5 | 180,8 | 99,6 | 155,9 | |
| 19 | Liver | 2009-12-11 | 45 | 3 | 29,2 | 64,7 | 60,7 | 10,2 | 54,2 | 57,7 | 204,5 | 183,7 | 13,7 | 152,1 | |
| 20 | Lung right | 2018-10-29 | 50 | 5 | 58,1 | 75,0 | 69,5 | 45,3 | 60,5 | 125,8 | 187,6 | 166,0 | 86,3 | 133,9 | |
| 20 | Lymph node mediastinum right | 2018-10-29 | 40 | 5 | 47,9 | 60,7 | 56,7 | 34,8 | 49,1 | 93,9 | 134,3 | 121,1 | 59,1 | 97,2 | |
| 21 | Lymph node left common iliac artery | 2015-03-12 | 45 | 10 | 19,1 | 67,8 | 58,2 | 15,7 | 54,1 | 22,8 | 113,8 | 92,0 | 18,1 | 83,3 | |
| 23 | Lymph node left common iliac artery | 2017-02-01 | 50 | 10 | 63,3 | 75,1 | 72,7 | 43,5 | 66,1 | 103,5 | 131,6 | 125,5 | 62,5 | 109,9 | |
| 24 | Lung right | 2017-04-26 | 30 | 5 | 33,6 | 41,5 | 38,2 | 23,0 | 36,9 | 56,1 | 75,8 | 67,5 | 33,6 | 64,2 | |
| 25 | Lymph node left common iliac artery | 2010-04-30 | 45 | 3 | 59,7 | 67,5 | 65,8 | 42,4 | 60,0 | 178,7 | 219,4 | 210,1 | 102,3 | 180,2 | |
| 26 | Lung left | 2011-06-03 | 45 | 3 | 49,3 | 74,5 | 65,9 | 41,0 | 59,1 | 130,5 | 259,6 | 210,4 | 97,0 | 175,8 | |
| 27 | Lung left | 2019-04-02 | 45 | 3 | 52,9 | 67,5 | 63,3 | 38,7 | 57,8 | 146,1 | 219,4 | 196,7 | 88,5 | 169,0 | |
| 28 | Lymph node right iliaca bifurcation | 2020-05-05 | 40 | 5 | 56,6 | 59,6 | 58,7 | 40,6 | 53,1 | 120,7 | 130,6 | 127,6 | 73,6 | 109,5 | |
| 29 | Lung right | 2022-03-07 | 45 | 3 | 53,2 | 67,7 | 63,4 | 40,3 | 59,3 | 147,4 | 220,4 | 197,6 | 94,5 | 176,6 | |
| 29 | Liver | 2022-03-07 | 50 | 5 | 51,1 | 74,5 | 67,1 | 28,5 | 62,1 | 103,4 | 185,5 | 157,1 | 44,8 | 139,3 | |
| 30 | Tuber ischiadicum  right | 2019-11-05 | 40 | 5 | 45,5 | 47,8 | 46,8 | 39,4 | 45,5 | 86,9 | 93,5 | 90,5 | 70,4 | 86,8 | |
| 31 | Lymph node retrocrural | 2019-08-22 | 50 | 10 | 66,2 | 73,3 | 71,6 | 46,5 | 65,8 | 110,1 | 127,1 | 122,9 | 68,0 | 109,0 | |
| 32 | Adrenal gland right | 2021-12-16 | 50 | 5 | 68,0 | 73,5 | 72,0 | 47,6 | 65,9 | 160,7 | 181,5 | 175,8 | 92,8 | 152,7 | |
| 32 | Lung right | 2021-12-16 | 50 | 5 | 61,2 | 76,1 | 69,9 | 28,6 | 61,9 | 136,3 | 191,8 | 167,5 | 45,0 | 138,4 | |
| 33 | Costae five left | 2019-06-02 | 40 | 8 | 44,7 | 48,4 | 47,2 | 18,9 | 42,9 | 69,7 | 77,8 | 75,2 | 23,4 | 65,9 | |
| 34 | Lumbar vertebrae nr five | 2021-05-31 | 35 | 5 | 32,4 | 57,7 | 40,1 | 26,9 | 38,6 | 53,5 | 124,4 | 72,3 | 41,4 | 68,4 | |
| 36 | Os pubis right | 2021-08-06 | 40 | 5 | 52,6 | 60,2 | 58,0 | 33,0 | 52,9 | 107,9 | 132,7 | 125,4 | 54,8 | 108,8 | |
| 37 | Lung right | 2021-03-12 | 50 | 5 | 66,0 | 75,6 | 72,5 | 48,9 | 67,6 | 153,0 | 189,9 | 177,7 | 96,7 | 159,2 | |
| 38 | Lung right | 2020-03-24 | 50 | 5 | 67,3 | 74,8 | 72,4 | 47,8 | 66,9 | 157,8 | 186,6 | 177,1 | 93,5 | 156,3 | |
| 39 | Lymph node aorta and vena cava inferior | 2019-09-18 | 40 | 5 | 51,5 | 58,9 | 57,5 | 37,3 | 51,2 | 104,7 | 128,3 | 123,8 | 65,2 | 103,5 | |
| 40 | Subcutaneously right abdomen | 2019-10-17 | 40 | 5 | 42,5 | 60,1 | 57,7 | 21,3 | 51,2 | 78,6 | 132,4 | 124,2 | 30,4 | 103,7 | |
| 40 | Subcutaneously left abdomen | 2019-10-17 | 48 | 6 | 64,9 | 73,1 | 70,9 | 39,3 | 64,8 | 135,1 | 162,1 | 154,8 | 65,0 | 134,8 | |
| 41 | Lymph node para-iliacal | 2021-04-23 | 40 | 5 | 39,4 | 61,1 | 53,8 | 29,3 | 48,3 | 70,5 | 135,8 | 111,7 | 46,4 | 94,8 | |
| 42 | Lung right | 2020-10-16 | 45 | 3 | 58,4 | 67,5 | 63,6 | 41,8 | 56,7 | 172,1 | 219,2 | 198,4 | 100,2 | 164,1 | |
| 42 | Lung left | 2020-10-16 | 45 | 3 | 59,5 | 67,6 | 63,8 | 39,7 | 56,1 | 177,5 | 219,8 | 199,3 | 92,4 | 161,2 | |
| SBRT stereotactic body radiation, *Gy* Grey, *CTV* clinical planning target volume, *PTV* planning target volume, *BED* biological equivalent dose | | | | | | | | | | | | | | |  |

| **Supplementary Table 2** SBRT dose, local control rate and local progression-free survival | | | |
| --- | --- | --- | --- |
| Nr of lesions treated with SBRT *n* = 51 | **Local control (*n*)** | **Local progress (*n*)** | ***p*-value** |
| All cohort^a^ | 42 | 6 |  |
| CTV-mean-BED interval, Gy  65-168  169-210 | 22  20 | 2  4 | 0.383 |
| PTV-mean-BED interval, Gy  55-146  147-183 | 22  19 | 2  4 | 0.352 |
|  | **HR local progression-free survival** | **95% CI** | ***p*-value** |
| **CTV-mean-BED (169-210 Gy ref.)**  65-168 Gy | 0.53 | 0.10-2.89 | 0.461 |
| **PTV-mean-BED (147-183 ref.)**  55-146 | 0.51 | 0.09-2.80 | 0.441 |
| ^a^Three patients missing local control data  SBRT stereotactic body radiation, *CTV* clinical planning target volume, *BED* biological equivalent dose, *Gy* Grey, *PTV* planning target volume | | | |

| **Supplementary Table 3** Univariable CoxPH analysis of PFS and OS | | | | | | |
| --- | --- | --- | --- | --- | --- | --- |
|  | **PFS** | | | **OS** | | |
| **Variable** | **HR** | **95% CI** | ***p-*value^a^** | **HR** | **95% CI** | ***p-*value^a^** |
| **Sex (male ref.)** |  |  |  |  |  |  |
| Female | 1.11 | 0.45-2.75 | 0.820 | 1.24 | 0.49-3.12 | 0.645 |
| **Age (54-67 years ref.)** |  |  |  |  |  |  |
| 68-75 years | 0.90 | 0.37-2.16 | 0.807 | 0.97 | 037-2.60 | 0.972 |
| 78-88 years | 0.81 | 0.34-1.95 | 0.634 | 1.24 | 0.49-3.12 | 0.651 |
| **ECOG performance status (ECOG 0 ref.)** |  |  |  |  |  |  |
| ECOG 1 | 0.85 | 0.40-1.81 | 0.678 | 1.52 | 068-3.37 | 0.306 |
| ECOG 2 | 2.77 | 0.62-12.44 | 0.183 | 2.54 | 0.55-11.76 | 0.234 |
| **Primary tumor (upper tract ref.)** |  |  |  |  |  |  |
| Bladder | 1.06 | 0.50-2.23 | 0.878 | 0.87 | 0.41-1.85 | 0.714 |
| **Primary curative treatment (yes ref.)** |  |  |  |  |  |  |
| Primary metastatic disease | 1.21 | 0.42-3.48 | 0.721 | 1.21 | 0.42-3.48 | 0.721 |
| **Palliative chemotherapy prior to SBRT (no ref.)** |  |  |  |  |  |  |
| Yes | 0.69 | 0.31-1.52 | **0.036** | 0.87 | 0.40-1.90 | 0.731 |
| **Metastatic site of SBRT (local recurrence ref.)** |  |  |  |  |  |  |
| Lymph node | 1.56 | 0.19-13.01 | 0.679 | 1.28 | 0.15-11.01 | 0.824 |
| Bone | 2.50 | 0.28-22.60 | 0.414 | 2.33 | 0.24-22.79 | 0.466 |
| Liver | 4.23 | 0.38-47.39 | 0.242 | 3.22 | 0.29-36.14 | 0.343 |
| Lung | 2.33 | 0.31-17.75 | 0.415 | 1.82 | 0.24-14.00 | 0.567 |
| Other | 2.62 | 0.27-25.24 | 0.406 | 2.46 | 0.27-22.21 | 0.423 |
| **Nr of metastasis treated at first SBRT (1 ref.)** |  |  |  |  |  |  |
| 2 or more | 4.12 | 1.81-9.38 | **0.001** | 2.08 | 0.91-4.76 | 0.084 |
| ^a^Log-rank (Mantel-Cox)  *CoxPH* Cox-proportional hazards regression, *PFS* progression-free survival, *OS* overall survival, *HR* hazard ratio, *CI* confidence interval, *ECOG* Eastern Cooperative Oncology Group. | | | | | | |
